# Single-cell mapping of human endometrium and decidua reveals epithelial and stromal contributions to fertility

**DOI:** 10.1172/jci.insight.195254

**Published:** 2026-01-23

**Authors:** Gregory W. Burns, Emmanuel N. Paul, Manisha Persaud, Qingshi Zhao, Rong Li, Kristin Blackledge, Jessica Garcia de Paredes, Pratibha Shukla, Ripla Arora, Anat Chemerinski, Nataki C. Douglas

**Affiliations:** 1 Department of Obstetrics, Gynecology and Reproductive Biology, Michigan State University, Grand Rapids, Michigan, USA.; 2 Department of Obstetrics, Gynecology and Reproductive Health, Rutgers Health, New Jersey Medical School, Newark, New Jersey, USA.; 3 Reproductive Medicine Center, The Affiliated Drum Tower Hospital of Nanjing University Medical School, Nanjing, China.; 4 Department of Pathology and Laboratory Medicine, Rutgers Health, New Jersey Medical School, Newark, New Jersey, USA.; 5 Department of Obstetrics, Gynecology and Reproductive Biology, Institute for Quantitative Health Science and Engineering, Michigan State University, East Lansing, Michigan, USA.; 6 Center for Immunity and Inflammation, Rutgers Health, New Jersey Medical School, Newark, New Jersey, USA.

**Keywords:** Cell biology, Reproductive biology, Fertility, Transcriptomics

## Abstract

The human endometrium undergoes dynamic changes across the menstrual cycle to establish a receptive state for embryo implantation. Using bulk and single-cell RNA-Seq, we characterized gene expression dynamics in the cycling endometrium and the decidua from early pregnancy. We demonstrated that during the mid-secretory phase — the period encompassing the window of implantation — secretory glandular epithelial cells undergo notable transcriptional changes and alterations in cell-cell communication. Through comprehensive analyses, we identified the glandular epithelium receptivity module (GERM) signature, comprising 556 genes associated with endometrial receptivity. This GERM signature was consistently perturbed across datasets of endometrial samples from women with impaired fertility, validating its relevance as a marker of receptivity. In addition to epithelial changes, we observed shifts in stromal cell populations, notably involving decidual and senescent subsets, which also play key roles in modulating implantation. Together, these findings provide a high-resolution transcriptomic atlas of the receptive and early pregnant endometrium and shed light on key molecular pathways underlying successful implantation.

## Abstract 2

This study reveals critical endometrial gene expression shifts, identifying a unique epithelial receptivity signature and stromal changes that support embryo implantation, and highlighting fertility-related disruptions.

## Introduction

Only 30%–40% of ovulatory human menstrual cycles result in spontaneous pregnancy (1). Coordinated actions of ovarian-derived estradiol and progesterone orchestrate changes in the cellular compartments of the endometrium, including the stromal, epithelial, immune, and endothelial cells (2), to prepare the endometrium for pregnancy. During the follicular phase of the menstrual cycle, a dominant follicle secretes increasing amounts of estradiol, eventually reaching the threshold required to initiate the luteinizing hormone surge and trigger ovulation (3–5). After ovulation, progesterone secreted from the corpus luteum exerts its actions on the endometrium, resulting in a differentiated tissue capable of embryo implantation (6). A brief period of endometrial receptivity follows; it is aptly termed the window of implantation and occurs during the mid-secretory phase between 6 and 10 days after the luteinizing hormone surge (7). This temporally restricted window is characterized by global changes in gene expression and an increase in secretory and metabolic activity (8). Attachment of the blastocyst-stage embryo to the luminal epithelium is followed by a cascade of events including embryo-derived trophoblast cell invasion of the endometrial stroma and spiral arterioles (9). Thus, pregnancy initiation depends on the successful crosstalk between a competent embryo and a receptive endometrium, which is defined by the cellular, molecular, and structural milieu. Abnormalities in endometrial or embryonal development, or dyssynchrony between these two elements, could result in implantation failure or pregnancy loss early in gestation (10).

Assisted reproductive technology (ART) is commonly used to overcome infertility. With in vitro fertilization and preimplantation genetic testing, embryos can be assessed for quality and ploidy to identify those with the highest chance of success. Studies comparing the endometrium of women in natural menstrual cycles and those undergoing ovarian stimulation have sought to identify factors associated with an implantation-competent secretory phase endometrium (11–13). Ovarian stimulation induces a dyssynchrony between endometrial glands and stroma, high levels of differentially expressed genes, and significant changes in the immune cell compartment (13). For this reason, embryo transfer in ART cycles is most often performed separately from ovarian stimulation (14). However, despite advances in ART, implantation rates only approach 70% in the most favorable conditions (15). Additionally, recurrent implantation failure, defined as failure to achieve pregnancy after several embryo transfers, affects up to 10% of individuals undergoing in vitro fertilization and embryo transfer (16, 17). Failed implantation suggests that the optimal endometrial environment has not been fully elucidated, underscoring the need for improved methods to assess and identify an endometrium receptive to embryo implantation.

Current methods to clinically assess endometrial receptivity are limited. In practice, transvaginal ultrasound evaluation of endometrial thickness is the widely accepted approach. In ART cycles, implantation rates are lower when endometrial thickness is less than 7 mm (18). However, the cellular, molecular, and structural changes that drive endometrial thickness and define a receptive endometrium are unknown. To address this, endometrial sampling in nonconception cycles, followed by histological and/or transcriptomic analysis, has been explored (19). It has been proposed that the window of implantation may have an mRNA signature that can be assessed using transcriptomics technologies. The endometrial receptivity array (ERA) was developed to determine the optimal timing for embryo transfer based on the expression of 238 specific receptivity-associated genes (20) in an endometrial biopsy sample obtained during a mock nonconception cycle. The ERA results, which describe the endometrium as prereceptive, receptive, or postreceptive, could require altering the timing of the subsequent embryo transfer to align with the most receptive day in the cycle. And thus, identification of the gene signature associated with a receptive endometrium, in conjunction with transvaginal ultrasound, would improve pregnancy rates. However, this tool has not been found to improve pregnancy rates for some groups, and recent RCTs evaluating the effectiveness of the ERA have yielded conflicting results (21–23).

Several gaps remain in our understanding of what constitutes an implantation-competent endometrium. First, it is unclear how gene expression varies throughout the secretory phase or from cycle to cycle in one individual. In addition to the absolute levels of gene expression, capturing the directionality of changes in gene expression is important. Second, bulk RNA-Seq analysis of human endometrial biopsies has presented inconsistent results across different studies. Bulk RNA-Seq also precludes cell type–specific analysis of gene expression. With the advancement of single-cell RNA-Seq (scRNA-Seq), it is becoming increasingly clear that endometrial cell lineages and subtypes exhibit distinct transcriptomic patterns (24–26). Therefore, cell subtypes, their transcriptomic changes, and cell-cell communication may play important roles in endometrial receptivity. Finally, the ERA was developed on a non-Hispanic White population. The broad applicability of the ERA to women from different races/ethnicities is lacking. Thus, a deeper understanding of the dynamic changes in the cycling endometrium is essential and will lay the groundwork for developing clinical tools capable of accurately predicting endometrial receptivity and reducing the risk of implantation failure.

To address these gaps, we performed bulk mRNA-Seq and scRNA-Seq analyses of endometrial samples obtained from healthy, fertile Black and Hispanic women across the menstrual cycle, as well as from first trimester decidual tissue. Our goal was to better understand stromal and epithelial cell changes that underlie endometrial differentiation in preparation for embryo implantation, with a focus on the acquisition of endometrial receptivity and the processes of decidualization and cellular senescence. Herein, we identify a critical role for the secretory glandular epithelium in supporting endometrial receptivity during the window of implantation. To our knowledge, this is the first human transcriptomic evidence supporting a critical role for the secretory glandular epithelium in endometrial receptivity, consistent with prior observations demonstrating the necessity of a functional glandular epithelium for fertility in mice and domestic animals (27–31). Moreover, we underscore the significance of this cell type in fertility across diverse populations.

## Results

### Experimental study design.

**Figure 1:**
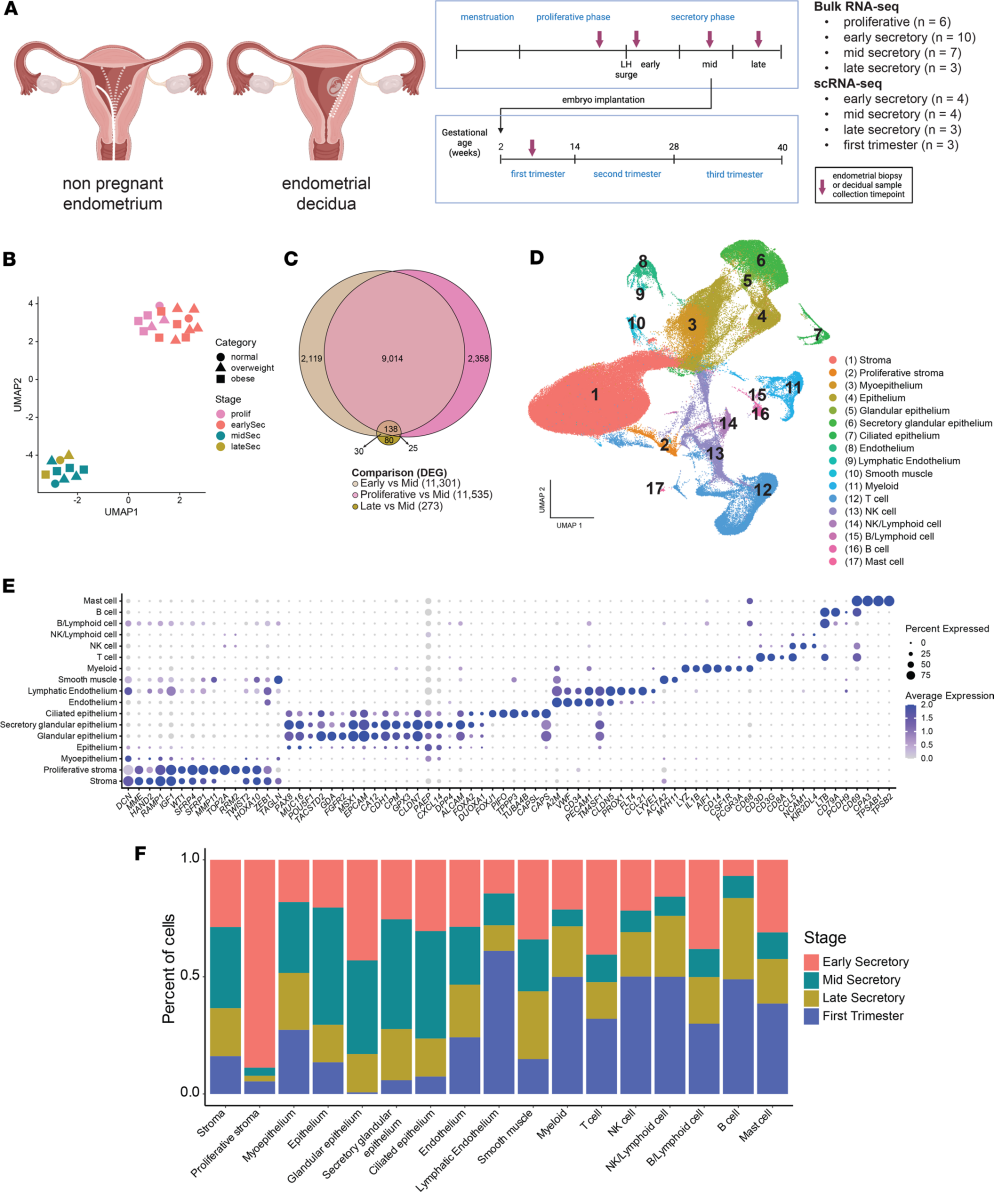
Identification of the transcriptomic profile and dynamic cell populations in the endometrium over time. (**A**) Summary of sample collection. (**B**) UMAP visualization of mRNA-Seq from proliferative (*n* = 6), early (*n* = 10), mid- (*n* = 7), and late (*n* = 3) secretory phase human endometrial samples. The color of each symbol indicates menstrual cycle stage; the shape denotes BMI category (kg/m2): normal (18–24.9), overweight (25–29.9), and obese (≥ 30). (**C**) Euler plot of the DEGs from proliferative versus mid-secretory, early versus mid-secretory, and late versus mid-secretory phase endometrial samples. (**D**) UMAP visualization of 171,261 isolated cells from human endometrial samples (*n* = 14). Each cluster (*n* = 17) represents a cell population with a similar transcriptomic profile. (**E**) Dot plot for cluster identification using specific markers for cell types from the endometrium. Average gene expression and percentage of cells expressing the specific gene in each cell cluster are shown by the color intensity and the diameter of the dot, respectively. (**F**) Stacked bar plot showing the proportion of cells in each cluster by stage.

The endometrium undergoes significant cellular changes across the menstrual cycle, particularly during the secretory phase, which follows ovulation and prepares the endometrium for embryo implantation. With pregnancy, the postimplantation endometrium is referred to as the endometrial decidua (32). To understand temporal changes in the transcriptomic signature within endometrial cell populations, we enrolled women with regular ovulatory cycles and proven fertility and those in the first trimester of pregnancy (Figure 1A). After enrollment, 27 study participants were assigned to 1 of 4 groups that corresponded to endometrial biopsy collection in the proliferative (*n* = 6), early (*n* = 10), mid- (*n* = 8), or late secretory (*n* = 3) phase of the menstrual cycle. Three additional participants consented to the collection of endometrial decidua at the time of an elective termination of pregnancy (Supplemental Table 1; supplemental material available online with this article; https://doi.org/10.1172/jci.insight.195254DS1). Study participants were similar with respect to age, BMI, gravidity (number of prior pregnancies), and parity (number of live births) (Supplemental Table 1). Our cohort consisted of: 87% of participants self-identified as Black/African American (26/30) and 13% (4/30) as Hispanic. Participants in the study had a mean (SD) age of 30.2 (5.2) years and a mean (SD) BMI of 33.0 (8.4) kg/m^2^. The median (IQR) number of pregnancies was 3 (2, 4) with 2 (2, 3) live births. The mean (SD) estradiol level at the time of the biopsy was 160.9 (74.3) pg/mL in the early secretory phase, 169.0 (119.5) pg/mL in the mid-secretory phase, and 120.7 (12.3) pg/mL in the late secretory phase. As expected, the mean (SD)progesterone level was 1.1 (0.8) ng/mL in the early secretory phase, peaked at 11.5 (3.7) ng/mL in the mid-secretory phase, and declined to 5.3 (0.9) ng/mL in the late secretory phase.

### Bulk and single-cell RNA atlas.

To identify global changes in the endometrial transcriptome across the menstrual cycle, bulk mRNA-Seq was performed on 26 endometrial biopsy samples from the proliferative and secretory phases. We confirmed menstrual stage separation of the bulk samples with the endest package (33) for molecular staging of human endometrium (Supplemental Figure 1A). Uniform manifold approximation and projection (UMAP) resolved 2 clusters, with proliferative and early secretory phase samples grouping together and mid- and late secretory phase samples forming a second cluster, indicating a phase-dependent separation of transcriptomic profiles (Figure 1B). This observation was supported by the number of differentially expressed genes (DEGs) found between proliferative versus early secretory phase samples (*n* = 205 DEGs) compared with proliferative versus mid-secretory (*n* = 11,535 DEGs); early versus mid-secretory (*n* = 11,301 DEGs); and late versus mid-secretory (*n* = 273 DEGs) phase samples (Figure 1C and Supplemental Table 2–5). Additionally, over 9,000 DEGs were in common between the proliferative versus mid-secretory phase and early versus mid-secretory phase comparisons. Normal weight, overweight, and obese categories were represented across the menstrual cycle biopsy time points, with no clear segregation by BMI (Figure 1B).

We used scRNA-Seq to determine the distribution of endometrial cell types across the secretory phase (*n* = 11) and in the decidua from first trimester pregnancies (*n* = 3) (Supplemental Table 1). A total of 171,261 cells passed quality control with an average of 27,654 reads per cell. UMAP resolved 17 main cell clusters (Figure 1D). Cluster identities were assigned using the expression profiles of canonical markers for cell populations expected to be found in the nonpregnant human endometrium (Figure 1E). Cells were identified in all clusters across all groups, and the proportion of each cell type varied by stage (Figure 1F and Supplemental Figure 1B). We identified similar cell clusters in the decidua; however, there were notable differences in the proportions of each cell type when compared with the secretory phases. Proliferative stromal cells were most abundant in the early secretory endometrium (89% of the cluster), whereas lymphatic endothelium (61%) and 4 clusters of immune cells — myeloid (50%), NK (50%), NK/lymphoid (50%), and B cells (49%) — were most abundant in the endometrial decidua of pregnancy. Additionally, compared with the decidua, all immune cell populations were decreased in the secretory phase samples (early = 24%, mid = 7%, late = 22%, decidua = 42%). Taken together, the global transcriptome and the single-cell analysis demonstrated dynamic shifts in endometrial gene expression and cell composition across the menstrual cycle, with a marked transition from an early secretory to mid-secretory phase phenotype and an immune cell–dominated environment in early pregnancy. These findings highlight key molecular and cellular changes that may be critical for endometrial receptivity and successful embryo implantation.

### Expression of ESR1 and PGR in the epithelial and stromal compartments of the secretory phase endometrium and first trimester decidua.

**Figure 2:**
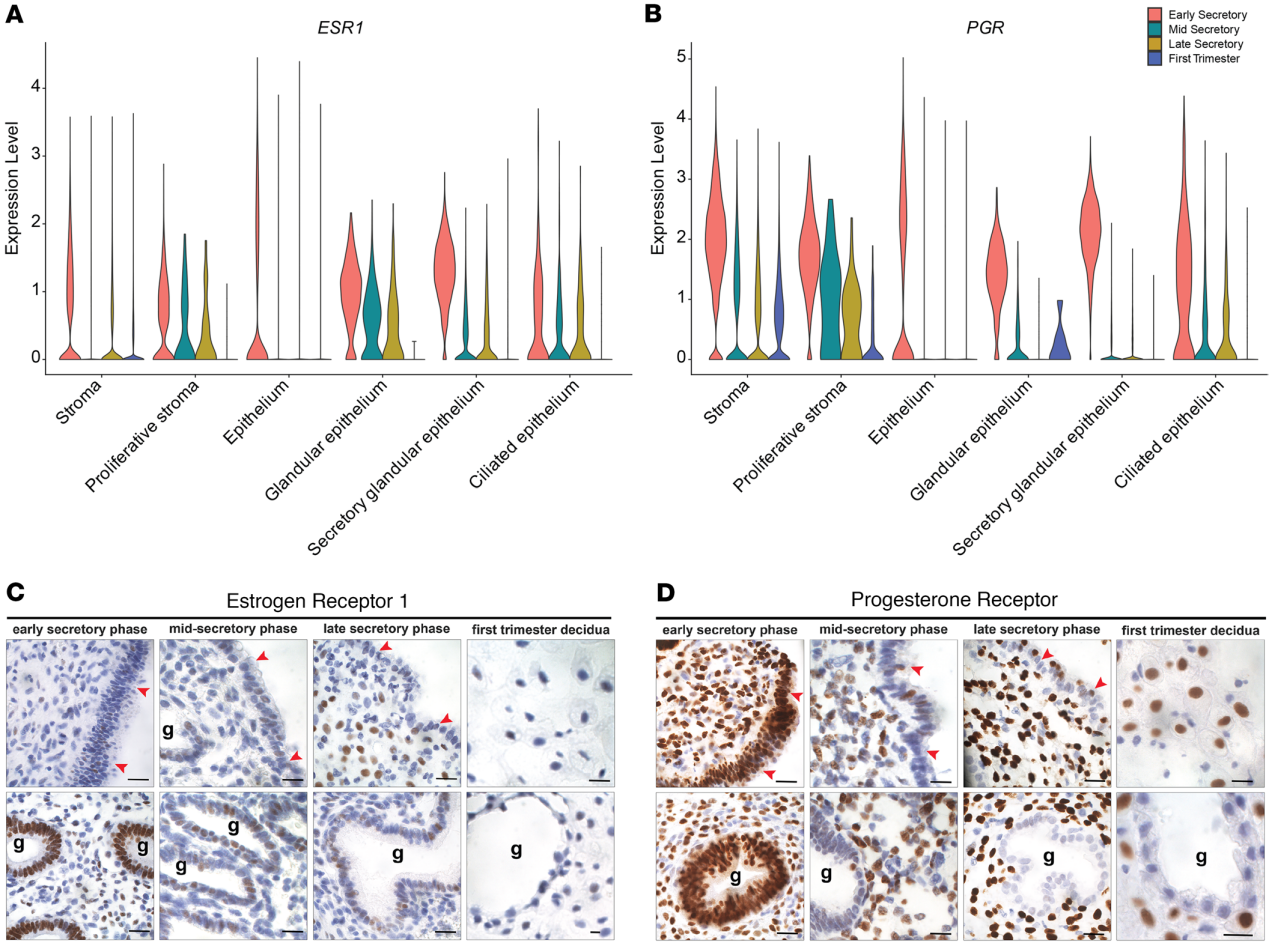
Expression of ESR1 and PGR in secretory phase endometrium and first trimester decidua. (**A** and **B**) Violin plots showing the expression of *ESR1* (**A**) and *PGR* (**B**) in the stromal and epithelial clusters across the secretory phase and in first trimester decidua. (**C** and **D**) Representative images of endometrial tissue sections stained to detect expression of ESR1 (**C**) and PGR (**D**) in glands and stroma of early, mid-, and late secretory phase endometrium and endometrial decidua of first trimester pregnancy. G, gland; red arrowheads identify the luminal epithelium. Scale bars: 10 μm.

Ovarian-derived estradiol (E2) and progesterone (P4) bind to their cognate receptors, estrogen receptor alpha (ESR1) and progesterone receptor (PGR), respectively, which mediate hormonal signaling to prepare the uterine lining for embryo implantation by regulating cellular proliferation, differentiation, and receptivity (34, 35). Circulating E2 and P4 levels were measured at the time of endometrial sampling (Supplemental Table 1), and we recently showed histopathological alignment of endometrial glandular and stromal compartments to menstrual cycle stage when the day of endometrial sampling was based on detection of the urinary luteinizing hormone surge (13). To understand how circulating E2 and P4 could affect the endometrium, we defined the spatial and temporal changes in *ESR1* and *PGR* across stages of the menstrual cycle in the epithelium and stroma (Figure 2). *ESR1* expression was observed in both stromal and epithelial cells during the early secretory phase, with higher levels in the glandular and secretory glandular epithelium (Figure 2A). Compared with the early secretory phase, *ESR1* expression was reduced in the mid- and late secretory phases and became more restricted to the proliferative stroma and glandular epithelium. These findings were corroborated at the protein level (Figure 2C). Similarly, *PGR* was highly expressed in both stromal and epithelial cells during the early secretory phase (Figure 2B) but showed a marked decrease in the mid- and late secretory phases, with very low expression in the epithelium. This pattern was also supported at the protein level (Figure 2D). These data confirm the dynamic regulation of ESR1 and PGR expression, highlighting a shift from widespread expression in the early secretory phase to a more localized and reduced expression pattern in the mid- and late secretory phases. This transition likely reflects the endometrium’s preparation for implantation and its progression toward a refractory state with changes in hormonal responsiveness. Moreover, comparison with the first trimester decidua revealed continued expression of PGR in the stroma and a further reduction in epithelial *ESR1* and *PGR* expression (Figure 2, C and D).

### ERA genes are expressed in the glandular epithelium.

**Figure 3:**
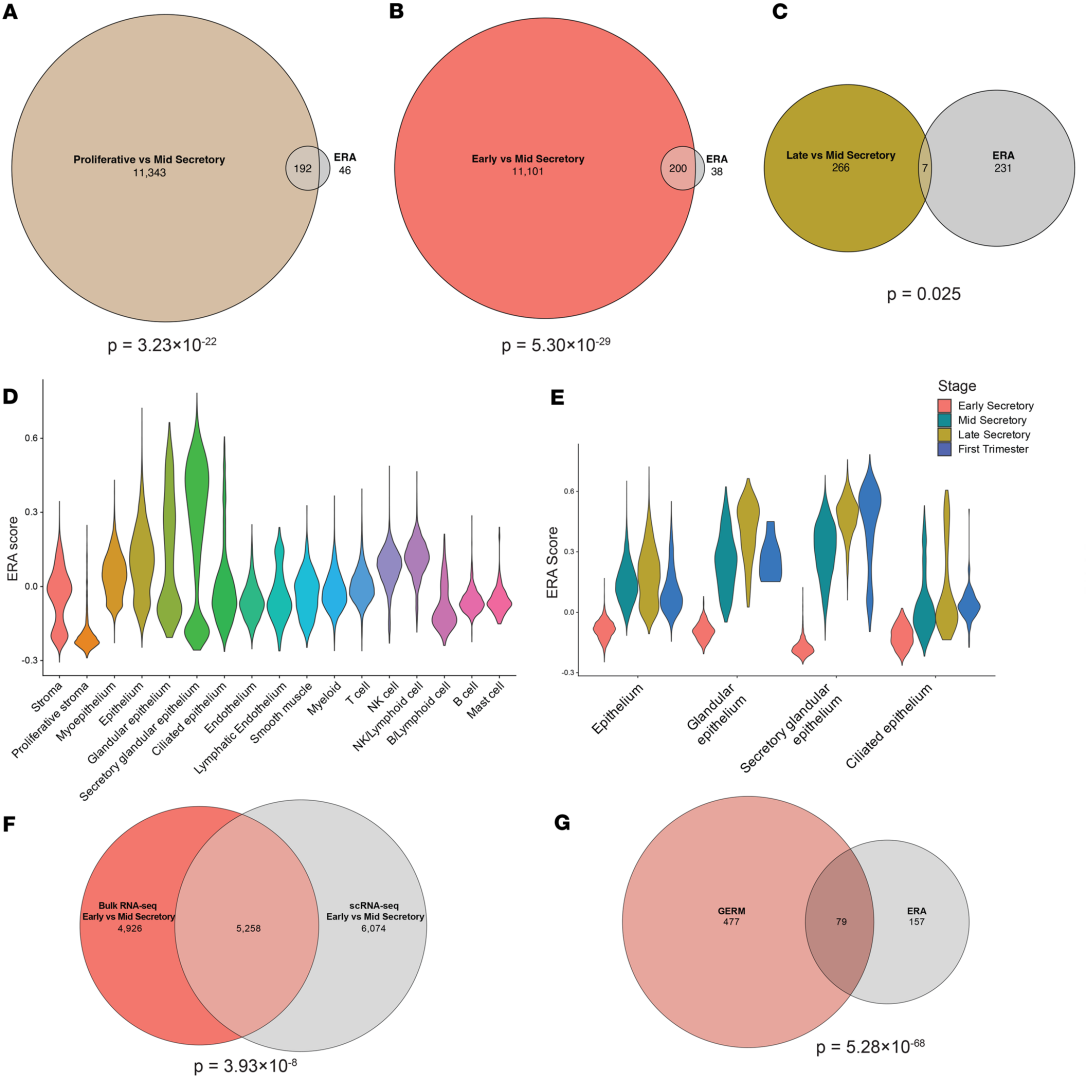
Endometrial receptivity array gene expression in bulk mRNA-Seq and scRNA-Seq analyses. (**A**–**C**) Euler plots of genes with significantly different expression in the proliferative versus mid-secretory (**A**), early versus mid-secretory (**B**), and late versus mid-secretory (**C**) phases as compared with the ERA. Hypergeometric test *P* values are indicated for each comparison. (**D** and **E**) Violin plots of the ERA score in each cell cluster (**D**) and the epithelium clusters across stages (**E**). (**F**) Overlap of DEGs from bulk and scRNA-Seq secretory glandular epithelium comparing early to mid-secretory endometrium. (**G**) The GERM score genes include 79 (33%) of the ERA genes.

The expression pattern of 238 genes defines the ERA (20). Analysis of gene expression patterns in our bulk mRNA-Seq data demonstrated that 84% of ERA genes were differentially expressed, in a consistent direction, from early compared with mid-secretory phase samples (132 increased, 68 decreased) and only 3% (4 increased, 3 decreased) overlapped with DEGs from mid- compared with late secretory phase samples (Figure 3, A–C). These findings confirmed expression of ERA genes in the mid-secretory phase. Next, utilizing an ERA score, which was defined by the 143 genes that are upregulated in the ERA (20), we used scRNA-Seq analysis of the endometrial samples to identify cell types corresponding to the receptivity signature. Glandular epithelium and secretory glandular epithelium had the highest expression levels of ERA genes (Figure 3D). The ERA marker genes had the lowest expression in the early secretory stage, with an overall increase at the mid-secretory phase and a continued rise in expression in the secretory glandular epithelium of late secretory phase and first trimester decidua samples (Figure 3E). Taken together, these data suggest that while the ERA gene signature is enriched in the mid-secretory phase, its expression extends beyond the traditional implantation window, persisting into the late secretory phase and early pregnancy. This finding highlights the power of scRNA-Seq for understanding temporal changes in gene expression within distinct epithelial cell types.

Based on the dispersion of the epithelial cluster in the UMAP analysis, we performed a subcluster analysis. In addition to glandular, secretory glandular, and ciliated epithelium, we identified 7 epithelial subclusters (Supplemental Figure 2A). The proportion of cells within each cluster varied dynamically, with an expansion of cells in the epithelium 0 subcluster from the early secretory phase to later stages and a decline of cells in glandular and secretory glandular epithelium in first trimester decidua samples (Figure 2D). These findings reveal stage-specific epithelial remodeling, potentially driven by shifts in cellular composition or dynamic transcriptional states. To further characterize these epithelial subpopulations, we defined the top marker genes for each subcluster (Supplemental Table 6), including a subcluster enriched for *LGR5*, a luminal epithelial stem/progenitor population (36–39). A dot plot of representative markers (Supplemental Figure 2B) highlighted genes enriched in individual subclusters, and bar plots (Supplemental Figure 2, C and D) provide additional context of epithelial diversity within the endometrium.

To identify marker genes that could define a receptive transcriptomic signature of the mid-secretory endometrium, we identified DEGs in the secretory glandular epithelial cells in early versus mid-secretory samples. As shown in Figure 3F, 11,332 genes were differentially expressed (Supplemental Table 7) with 5,258 overlapping DEGs identified in bulk mRNA-Seq comparisons of early versus mid-secretory phases. We further filtered these overlapping DEGs using a cutoff of an absolute value of the log_2_ fold-change greater than 2 and an FDR less than 10^–7^ and found 556 marker genes (267 downregulated and 289 upregulated, Supplemental Table 8) that we termed the glandular epithelium receptivity module (GERM) signature. Notably, 33% of the ERA (*n* = 79) genes were included in the GERM signature (Figure 3G).

### In vitro decidualization and senescence marker genes are highly expressed in decidua from first trimester pregnancy.

**Figure 4:**
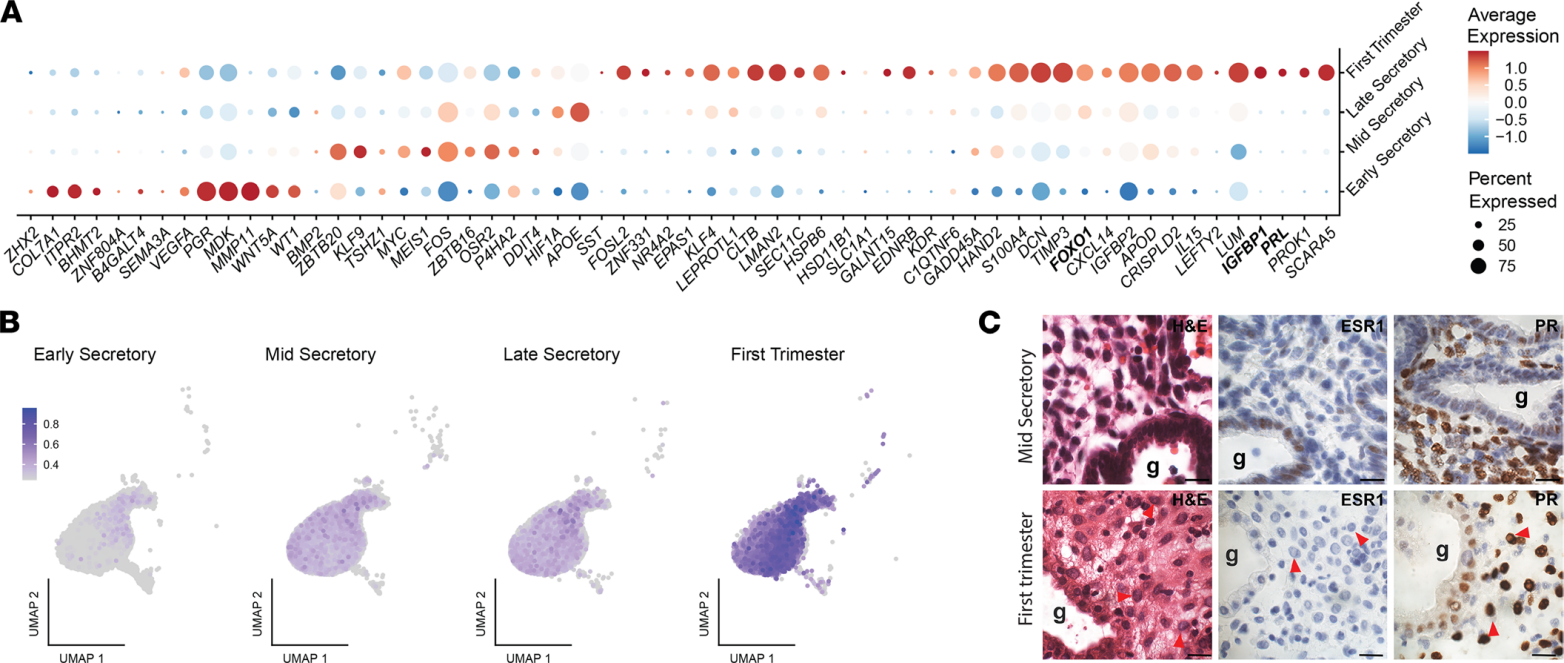
Markers of in vitro decidualization are highly expressed in endometrial decidua from the first trimester of pregnancy. (**A**) Dot plot of in vitro decidualization markers in the stroma cluster separated by stage. Expression of *IGFBP1*, *FOXO1*, and *PRL* is highlighted in bold. (**B**) UMAP of the in vitro decidualization marker score in the stroma cluster. Average score expression is represented by color intensity. (**C**) Representative images of tissue sections from the mid-secretory phase and first trimester decidua stained with H&E (scale bars: 10 μm). Red arrowheads are decidualized stromal cells with epithelioid morphology; g, gland.

In vivo, estrogen-primed endometrial cells differentiate under the influence of progesterone, thus generating an endometrium that is receptive to embryo implantation. Stromal cell decidualization, which is an integral part of endometrial remodeling, has been frequently studied, perturbed, or recovered in vitro to gain a deeper understanding of the mechanisms underlying appropriate versus aberrant endometrial differentiation. Decidualization of stromal cells in vitro is commonly verified by increased expression of “classic” markers of decidualization including *IGFBP1* (40), *PRL* (41), and *FOXO1* (42, 43). We combined these classic markers, as well as others associated with in vitro decidualization (44, 45) and found that expression of genes associated with in vitro decidualization was highest in the first trimester decidua when visualized across the secretory phase and first trimester samples (Figure 4A). The gene list was then used to generate a “decidualization score” to identify cells that expressed in vitro decidualization marker genes. Cells exhibiting high marker expression were predominantly found in the first trimester stromal cluster, with lowest expression observed during the early secretory phase compared with the mid- and late secretory phases (Figure 4B). Stromal cells with a rounded, epithelioid morphology and prominent nuclei, as is seen after in vitro decidualization of isolated primary or immortalized endometrial stromal cells (46), were detected by H&E staining in the first trimester but not in the mid-secretory phase (Figure 4C). These data demonstrate gene expression and the differentiated cellular morphology consistent with decidualization in stromal cells in first trimester deciduae.

**Figure 5:**
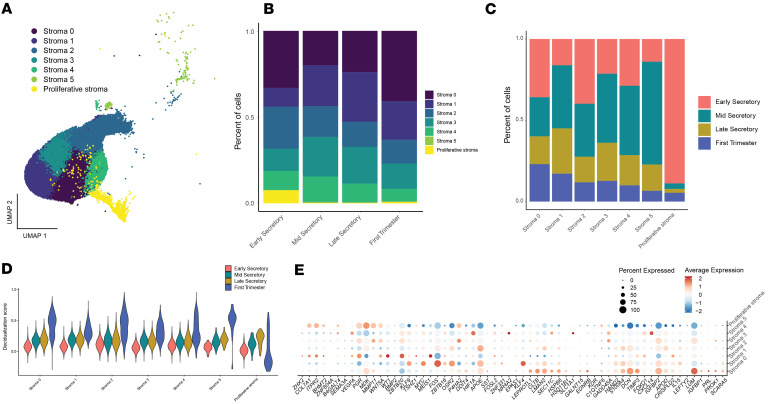
Stromal subcluster distribution and diffuse expression of in vitro decidualization markers in secretory phase endometrium and first trimester decidua. (**A**) UMAP of stroma subclustering showing 5 stroma subclusters in addition to the proliferative cluster. (**B**) Stacked bar plot of the stroma cells showing percentages of cells in the stroma subclusters by stage. (**C**) Stacked bar plot of the stroma cells showing percentages of cells for each stage by subcluster. (**D**) Violin plot of the decidualization score across the stroma subclusters split by stage. (**E**) Dot plot of in vitro decidualization markers in the stroma subclusters.

Cells with an elevated decidualization score were not uniformly distributed within the stromal cluster, supporting the utility of reclustering the stromal population for more detailed analyses. Six stromal subclusters were found in addition to the previously labeled proliferative stroma cluster (Figure 5A). The cell type proportions varied across stages, except stroma 4 (early = 11%, mid = 15%, late = 11%), with an increase from the early to mid-secretory and late secretory phases in stroma 1, 3, and 5 (stroma 1: early = 11%, mid = 24%, late = 29%; stroma 3: early = 13%, mid = 23%, late = 21%; stroma 5: early = 0.1%, mid = 0.6%, late = 0.3%). This corresponded to decreases in proliferative stroma and stroma 0 and 2 (proliferative: early = 8%, mid = 0.3%, late = 0.3%; stroma 0: early = 33%, mid = 20%, late = 24%; stroma 2: early = 24%, mid = 18%, late = 14%) (Figure 5, B and C). Analysis of in vitro decidualization markers in the subclusters confirmed higher expression in the first trimester stromal cells (Figure 5D). However, no specific cell population demonstrated consistently elevated expression of these in vitro decidualization markers (Figure 5, D and E).

### Senescent stromal cells are most abundant in the secretory phase endometrium and decidua from first trimester pregnancy.

**Figure 6:**
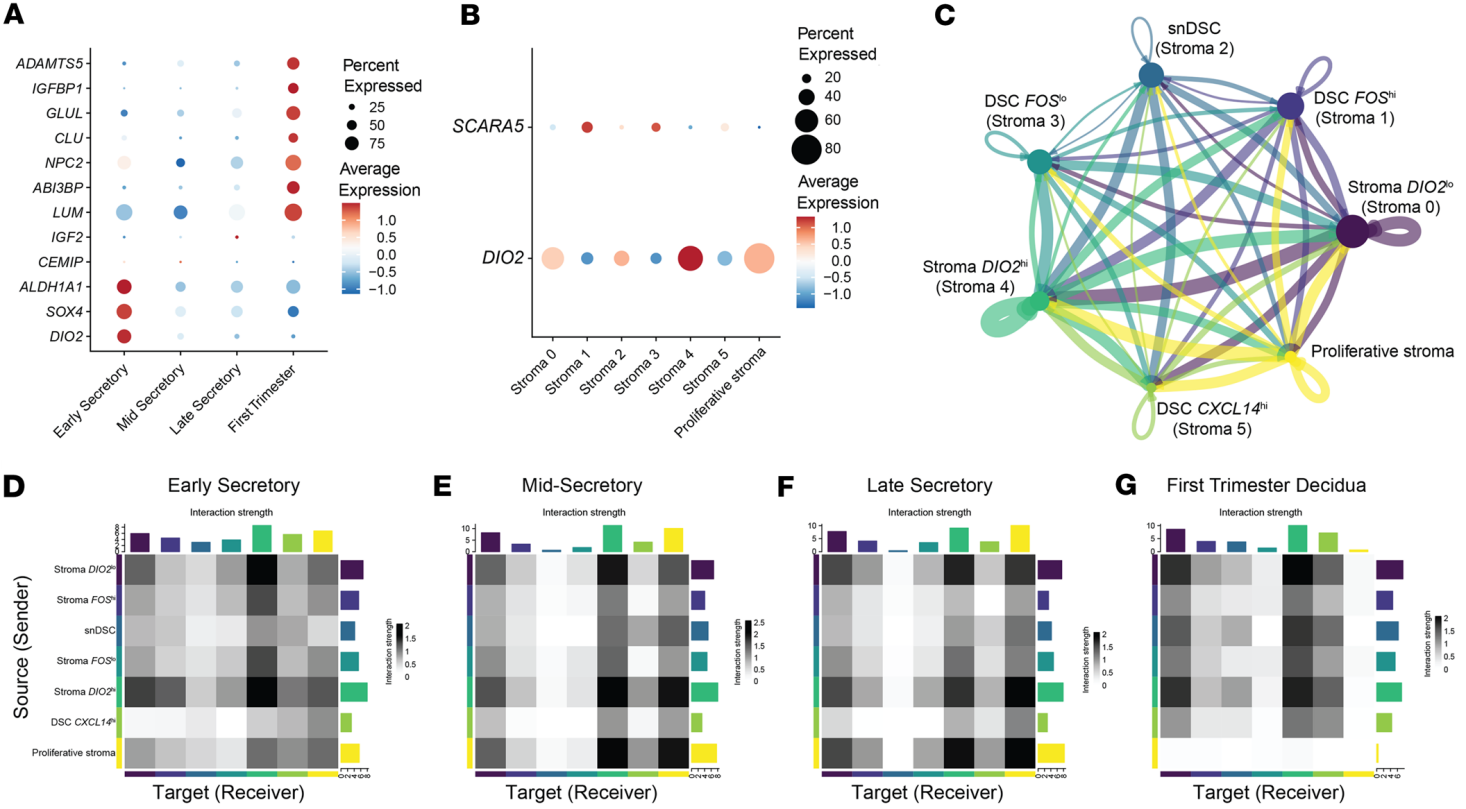
Cluster identification and communication in the stroma. (**A**) Dot plot of in vitro senescence markers across stages. (**B**) Dot plot of *SCARA5* and *DIO2*, in vivo markers for decidualization and senescence, respectively. (**C**) Cell-cell communication of the stroma clusters. Edge colors are consistent with the sources as sender, and edge weights are proportional to the interaction strength; that is, a thicker line indicates a stronger signal. Circle sizes are proportional to the number of cells in each cluster. Communication in the early secretory (**D**), mid-secretory (**E**), and late secretory (**F**) phases, and first trimester decidua of pregnancy (**G**) are shown as heatmaps. Colored bars represent the relative signaling strength of pathways across subclusters. The top-colored bar plot represents the sum of each column of the absolute values displayed in the heatmap (incoming signaling). The right colored bar plot represents the sum of each row of the absolute values (outgoing signaling).

Senescent stromal cells are present in secretory phase endometrium and likely play important roles in fertility (44). We utilized a panel of in vitro cellular senescence marker genes (44, 45) to identify senescent cells in our endometrial samples. We found 7 of the 12 genes had the highest expression in first trimester endometrial deciduae (Figure 6A), mirroring expression of in vitro decidualization marker genes, and that expression of in vitro senescence markers was not restricted to a single stroma subcluster (Supplemental Figure 3). We then used expression of validated in vivo markers, *SCARA5* and *DIO2* (44), to differentiate decidual and senescent subclusters. Stroma subclusters 1, 3, and 5, which were most abundant in the mid- and late secretory phase samples, had highest expression of *SCARA5* and were identified as decidualized stromal cells (DSCs) (Figure 6B). These were further characterized by expression of *FOS*, denoted as DSC *FOS*^lo^ and DSC *FOS*^hi^, or high expression of *CXCL14*, denoted as DSC *CXCL14*^hi^ (Figure 5E and Figure 6B). Stroma 0 and 4, with increased expression of *DIO2*, were identified as senescent stromal subclusters and were further characterized by the level of *DIO2* expression, denoted as *DIO2*^lo^ and *DIO2*^hi^ (Figure 6B). Stroma subcluster 2 expressed *SCARA5* and *DIO2* and was labeled senescent decidualized stromal cells (snDSCs) (Figure 6B).

To identify unique signaling patterns among stromal subclusters, ligand-receptor communication analysis was conducted using CellChat (47, 48). This analysis revealed that overall stromal communication was driven by senescent *DIO2*^lo^ and *DIO2*^hi^ cells (Figure 6C). In contrast, the snDSC cluster displayed the lowest overall signaling (Figure 6C). Assessment of stromal cell-cell communication across stages showed similar signaling patterns in mid- and late secretory phases with snDSCs having the lowest receiver score (Figure 6, E and F). Additionally, increased communication between the proliferative stroma and senescent *DIO2*^hi^ cells was evident during mid- and late secretory phases (Figure 6, E and F). Based on sender and receiver scores, senescent *DIO2*^hi^ and *DIO2*^lo^ and proliferative stroma cells were the most interactive clusters during early, mid-, and late secretory phases (Figure 6, D–F). In contrast, proliferative stroma was the least interactive subcluster in first trimester deciduae (Figure 6G).

### Stromal cell communication with secretory glandular epithelium increases during the mid-secretory phase.

The epithelial and stromal compartments of the endometrium have independent and interdependent roles in supporting embryo implantation (49, 50). While the epithelium undergoes critical modifications to establish receptivity for nidation (51, 52), stromal cell differentiation is essential for successful implantation and subsequent pregnancy maintenance (53). However, the mechanisms by which epithelial and stromal cells coordinate their functions during this process remain incompletely defined. To better understand the interactions between these two compartments, we defined the crosstalk between the epithelium and stroma cell populations.

**Figure 7:**
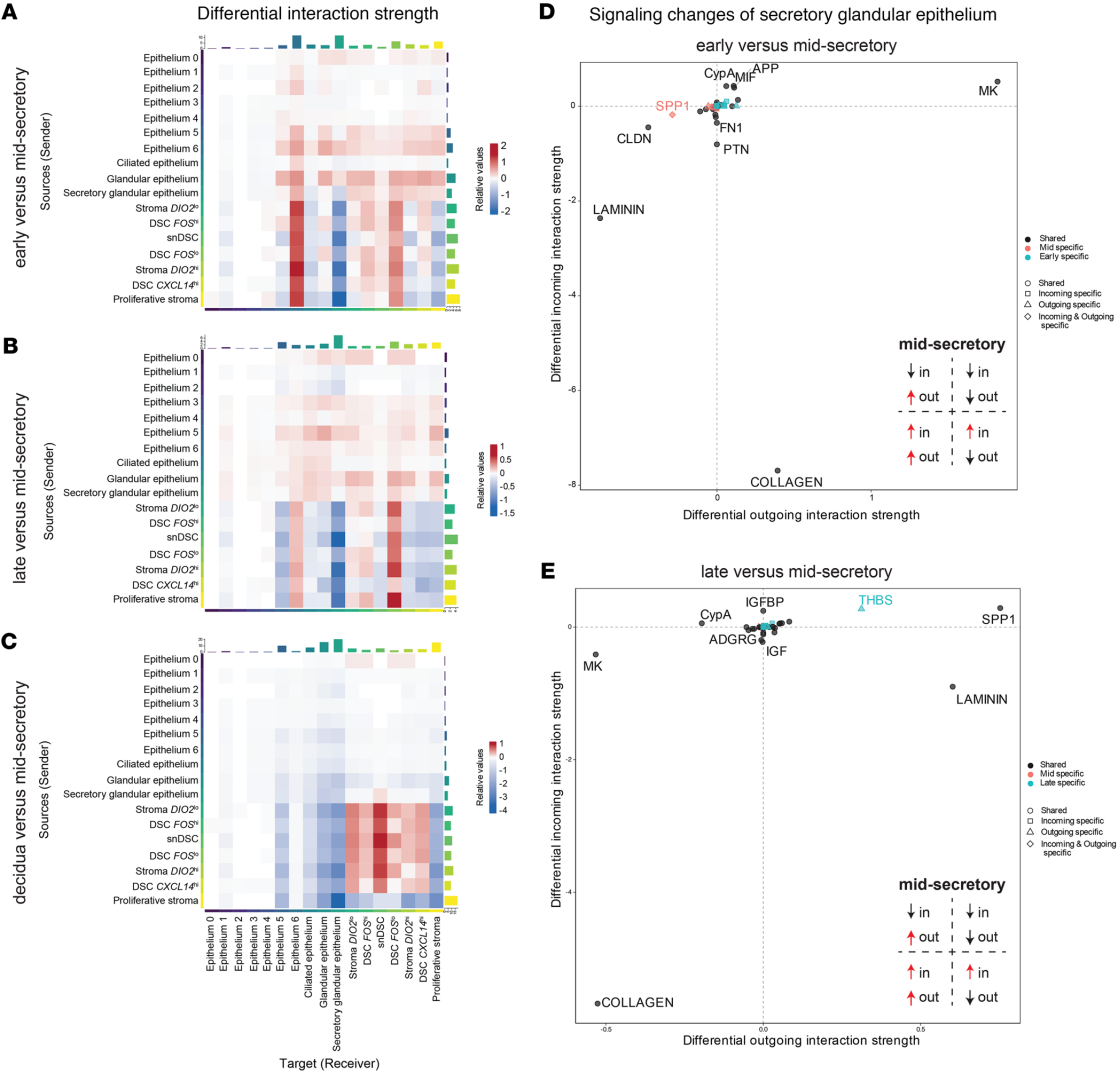
Stroma-epithelium communication peaks at mid-secretory phase in the secretory glandular epithelium. (**A**–**C**) Communication changes between the epithelium and stroma clusters in the early versus mid-secretory phase (**A**), late versus mid-secretory phase (**B**), and first trimester decidua versus mid-secretory phase (**C**) shown using heatmaps. Colors represent the relative signaling strength of a signaling pathway across clusters; blue indicates decreased communication and red an increase of communication probability. The top-colored bar plot represents the sum of the absolute values for each column displayed in the heatmap (incoming signaling). The right colored bar plot represents the sum of the absolute values of each row (outgoing signaling). (**D** and **E**) Signaling changes in the secretory glandular epithelium cluster are shown in scatter plots for early versus mid-secretory (**D**) and late versus mid-secretory (**E**).

Epithelial clusters 0 through 4 had the lowest receiver strength across all stages and had low interaction strengths. In the secretory phases, the glandular, secretory glandular, and all stroma subclusters were interactive (Figure 7, A–C). Epithelial cells exhibited reduced cell-cell communication in the first trimester decidua samples. In contrast, signaling between all stroma subclusters except for the proliferative stroma was active in the first trimester decidua samples (Figure 7C).

Epithelium 6 and DSC *FOS*^lo^ clusters had higher receiver strength in the early secretory compared with the mid-secretory phase, with stroma as the predominant source. By contrast, the stroma clusters had reduced communication with the secretory glandular epithelium in the early secretory phase samples (Figure 7A). A similar pattern was observed for late secretory compared with mid-secretory phase samples (Figure 7B), indicating that stroma to secretory glandular epithelium communication was increased during the mid-secretory phase. Conversely, communication from the stroma to epithelium 6 and DSC *FOS*^lo^ decreased in the mid-secretory phase samples (Figure 7, A and B). In the first trimester deciduae compared with mid-secretory phase samples, all stroma subclusters, except proliferative, had increased interaction strength within the stroma (Figure 7C). Interestingly, receiver strength for the snDSC cluster was increased in the first trimester decidua samples compared with mid-secretory phase samples.

**Figure 8:**
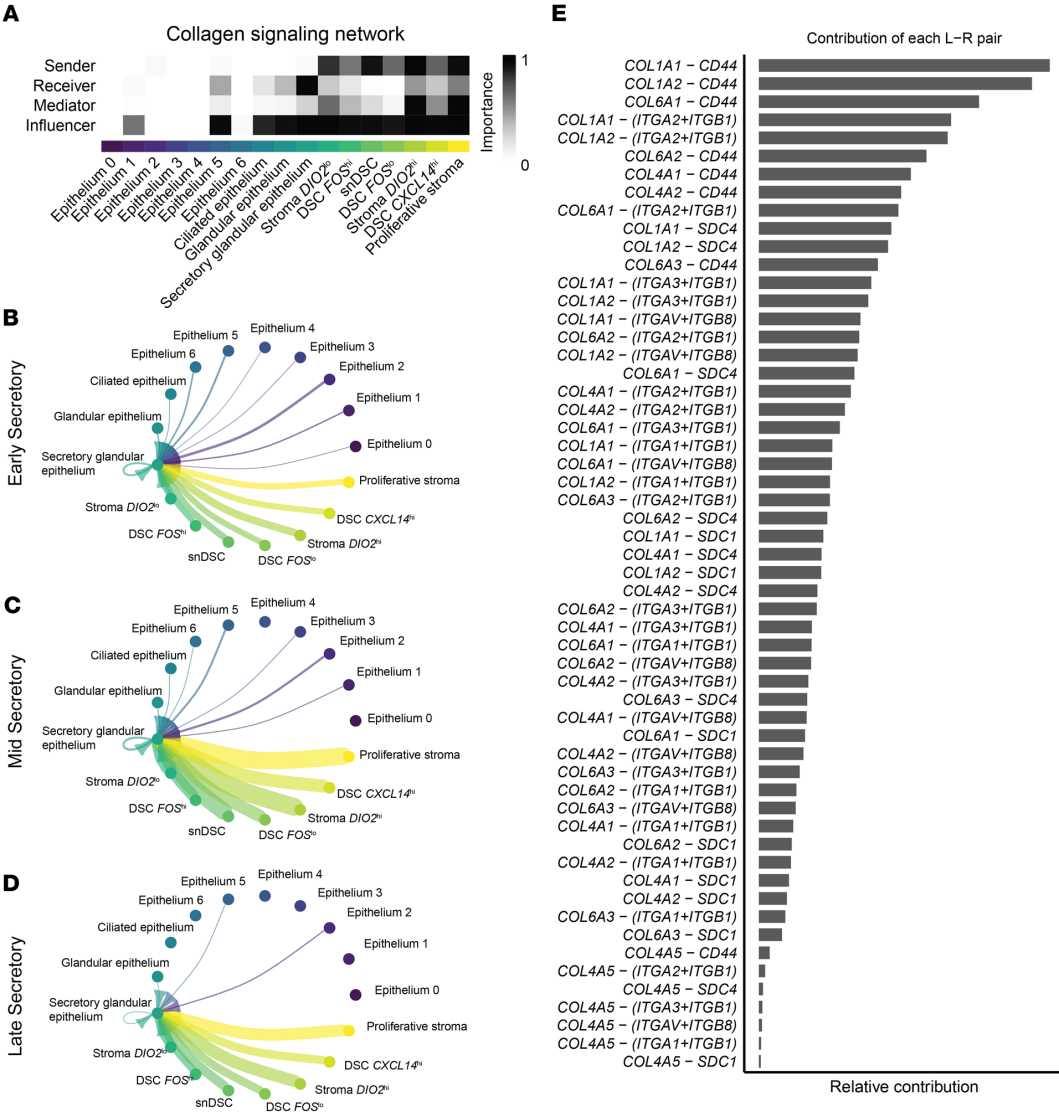
Collagen signaling between the stroma and epithelium during the secretory phase. (**A**) Role of each cell cluster in collagen signaling shown as a heatmap. (**B**–**D**) Cell-cell communication of the stroma and epithelium with the secretory glandular epithelium as the recipient. Edge colors are consistent with the sources as sender, and edge weights are proportional to the interaction strength; that is, a thicker line indicates a stronger signal. Circle sizes are proportional to the number of cells in each cluster for the early secretory (**B**), mid-secretory (**C**), and late secretory (**D**) phases. Note the increased communication from stroma subclusters during mid-secretory and late secretory phases. (**E**) Relative contribution of ligand-receptor pairs for signals coming into the secretory glandular epithelium.

Since the transition from the early to mid-secretory phase is critical for pregnancy initiation, we investigated altered signaling pathways in the secretory glandular epithelium, the primary cell type exhibiting changes in communication between the epithelium and stroma. Pathways associated with the extracellular matrix (ECM), including collagen and laminin, were downregulated in the secretory glandular epithelium of early and late secretory phase samples compared with mid-secretory phase samples (Figure 7, D and E). Signaling network analysis during the mid-secretory phase confirmed that the secretory glandular epithelium was the strongest signal recipient within the collagen pathway, receiving input from all stromal subclusters, as well as the epithelium 5 and ciliated epithelium subclusters (Figure 8A). The ligand-receptor interaction was strongest during the mid-secretory phase (Figure 8C) compared with the early (Figure 8B) and late secretory phases (Figure 8D), suggesting a key role for collagen signaling in endometrial receptivity. Further analysis of ligand-receptor interactions within the collagen pathway revealed that the primary ligand contributors were *COL1A1*, *COL1A2*, *COL6A1*, *COL6A2*, *COL4A1*, and *COL4A2*; *CD44* was the predominant receptor in the secretory glandular epithelium (Figure 8E).

### Dysregulated expression of epithelial cell– and stromal cell–specific genes in the endometrium of patients with infertility.

**Figure 9:**
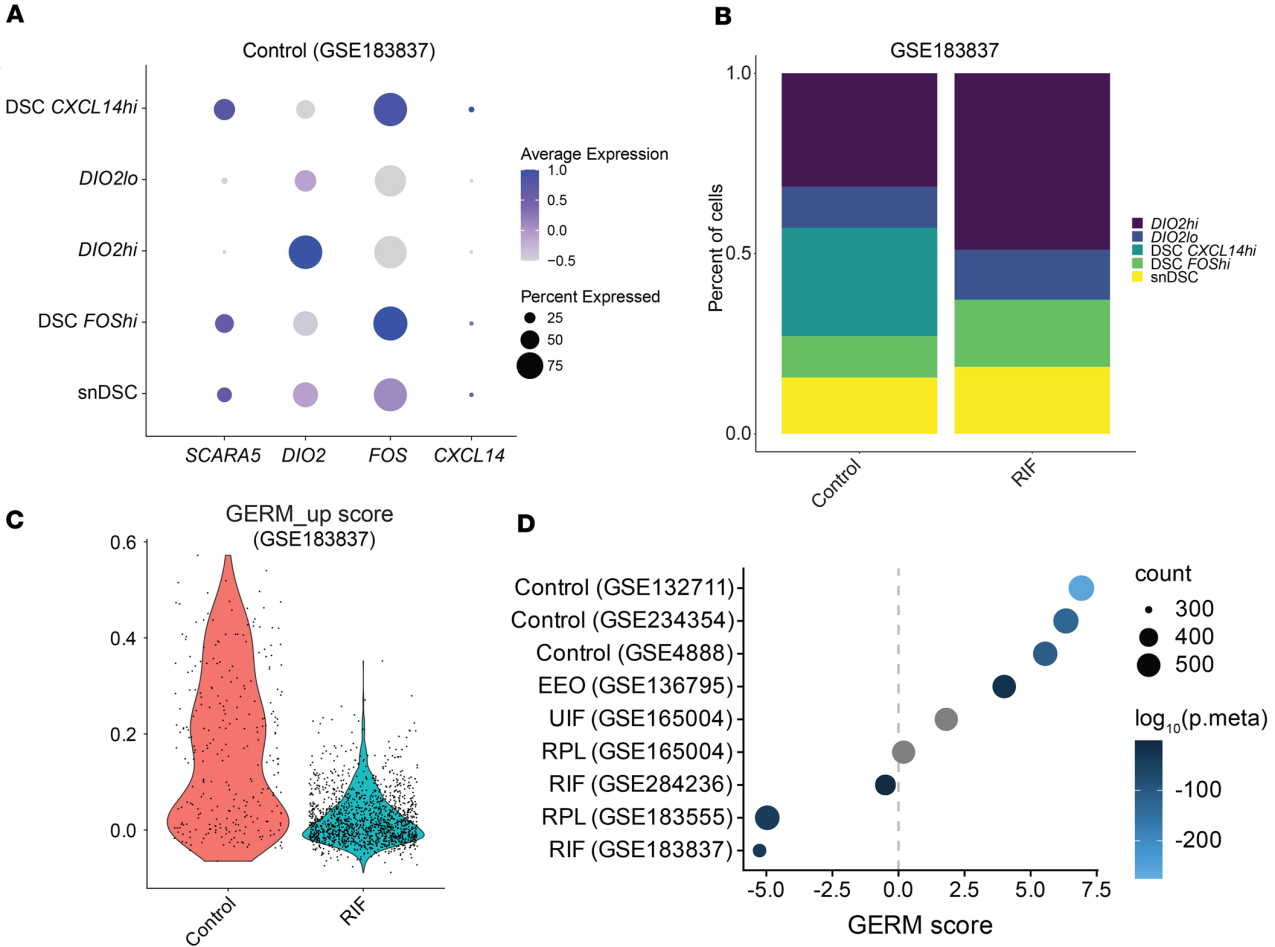
Disrupted stromal cell populations and epithelial gene expression in patients with infertility. (**A**) Dot plot of *SCARA5*, *DIO2*, *FOS*, and *CXCL14* for identification of the stroma populations in control endometrium samples from an scRNA-Seq dataset (GSE183837). (**B**) Stacked bar plot shows altered stroma cell proportions in endometrial samples from patients with recurrent implantation failure (RIF). (**C**) GERM upregulated genes in the secretory glandular epithelium are reduced in patients with RIF. (**D**) GERM score is positively correlated with mid-secretory endometrium from control patients (GSE132711, *n* = 11; GSE234354, *n* = 81; GSE4888, *n* = 11) and endometrial epithelial organoids (EEO; GSE136795, *n* = 6), but not from patients with unexplained infertility (UIF; GSE165004, *n* = 48), recurrent pregnancy loss (RPL; GSE165004, *n* = 48 and GSE183555, *n* = 10), or RIF (GSE284236, *n* = 16 and GSE183837, *n* = 9). A gray circle indicates an insignificant combined *P* value (p.meta) ≥ 0.05.

Based on the observed temporal increase in communication between stroma and secretory glandular epithelium during the mid-secretory phase, as well as the localization of genes associated with endometrial receptivity within the same epithelium, we hypothesized that this signaling axis would be altered in the endometrium of patients experiencing infertility. We first identified decidual and senescent stromal cells in a publicly available dataset of endometrial samples at the mid-secretory stage from controls and patients with recurrent implantation failure (GSE183837) (2). Stromal populations were classified in control samples based on our scRNA-Seq markers (Figure 9A), and we found disrupted proportions in endometrial samples from patients with recurrent implantation failure (Figure 9B). There was an expansion of senescent cells, expressing *DIO2*, and a concurrent decrease in decidual cell populations. Notably, *CXCL14*^hi^ decidual stromal cells were severely decreased in samples from patients with recurrent implantation failure compared with controls (0.2% vs. 30%).

We then applied our GERM signature to multiple datasets including those containing early- and mid-secretory phase endometrial samples from fertile controls (33, 54, 55) and patients with recurrent implantation failure (2, 56), recurrent pregnancy loss (57, 58), and unexplained implantation failure (57) (Figure 9, C and D). We confirmed reduced expression of the upregulated genes in our GERM signature (GERM_up) in the secretory glandular epithelium in endometrial samples from patients with recurrent implantation failure (Figure 9C). Gene set enrichment analyses using our GERM signature, GERM_up and GERM_down, were then combined to produce a GERM score for the other datasets, which included microarray, bulk RNA-Seq, scRNA-Seq, and spatial RNA-Seq results.

The GERM score was significantly higher and positively correlated with healthy endometrium during the mid-secretory compared with the early secretory phase (scores = 5.6–6.3; Figure 9D). Notably, epithelial isolates from fertile endometrium (GSE132711) (55) exhibited the highest GERM score (7.2) and the strongest significance (combined *P* = 2.52 × 10^–220^). Similarly, endometrial epithelial organoids treated with estradiol and medroxyprogesterone acetate, which mimic the secretory phase, also displayed a significant positive GERM score (score = 4) (59). Consistent with these findings, 303 genes (54.5% of the GERM set) were coordinately differentially expressed in the hormone-treated organoids. Moreover, a significant overlap was identified between the GERM signature and endometrial PGR cistrome genes (*n* = 653), with 86 genes likely representing direct targets of PGR-mediated transcriptional regulation.

Our bulk mRNA-Seq dataset, as a positive control, had a GERM score of 8 with a combined *P* value of 0. In contrast, GERM scores in endometrial samples from patients with infertility, specifically recurrent implantation failure, recurrent pregnancy loss, or unexplained implantation failure, were decreased (scores = –5.3–1.8). The GERM scores from one microarray study (GSE165004) (57) were reduced (scores = 0.2–1.8) but did not reach significance. Inspection of the fold-changes revealed a limited dynamic range that likely affected the strength of the gene set enrichment analyses. Together, these findings validate our GERM signature across control patients of different racial and demographic origins and demonstrate dysregulation of our GERM signature genes in patients with “endometrial factor” infertility. The GERM gene list expands the panel of genes associated with endometrial receptivity, specifically capturing those expressed in secretory glandular epithelial cells. Additionally, our findings demonstrated that the glandular epithelium gene signature was correlated with receptivity required for successful embryo implantation.

## Discussion

In this study, we performed bulk and single-cell transcriptomic analyses on endometrial samples from ovulatory menstrual cycles and deciduae from first-trimester pregnancies to elucidate the epithelial and stromal changes driving endometrial differentiation in preparation for implantation. To our knowledge, this is the first study to include fertile Black and Hispanic patients, addressing a critical gap in reproductive research and providing an important foundation for future studies involving larger and more diverse cohorts. We demonstrated that glandular epithelial cells are central to endometrial receptivity and characterized shifts in stromal decidual and senescent populations, highlighting the complex, multilineage endometrial changes that underlie embryo implantation. Importantly, the GERM signature we developed remained robust when validated across multiple independent datasets encompassing 190 patients. This consistency, along with the observation of similar gene expression patterns among fertile controls of different racial and demographic origins, suggests that the molecular signature defining the window of implantation is conserved across groups.

Estrogen receptors and progesterone receptors mediate hormone signaling pathways that are essential for regulating changes in the endometrium throughout the menstrual cycle in preparation for potential pregnancy. Comparison with prior studies demonstrated that ESR1 and PGR gene and protein expression (60, 61), as well as gene expression profiles in the epithelial compartment (33, 54), are consistent among fertile and presumably fertile patients with regular menstrual cycles. The distribution of ESR1 and PGR proteins in our secretory phase samples aligns with published literature, which indicates that ESR1 and PGR expression is highest in the proliferative phase and decreases as the menstrual cycle progresses (62–66).

Since at least the early 20th century, clinicians and scientists have been attempting to describe and characterize the features of an endometrium that is receptive to embryo implantation. In 1950, Noyes et al. put forth criteria for dating the endometrium based on phenotypic features of endometrial glands and stroma on histopathology (51), facilitating the uniform categorization of a biopsy sample as early, mid-, or late proliferative, and describing the secretory phase in terms of the number of days after ovulation. The Noyes criteria were later applied to the examination of luteal phase defects (67) and assessed for correlation with fertility status (68) but have been largely relegated to the realm of pathologists for descriptive purposes only. More recently, attempts were made to characterize the window of implantation based on a transcriptomic signature, a technique theorized to optimize embryo transfer timing by uncovering prereceptive and postreceptive endometria on the day of typical receptivity (20). Unfortunately, the 238-gene tool, known as the ERA, has not consistently improved pregnancy rates for some groups (21–23). Despite the limitations of the ERA in improving pregnancy rates, we were interested in examining markers of receptivity used in their analyses to understand the cellular origins of the receptivity signature and to explore the cellular and molecular factors that contribute to endometrial receptivity. In this study, we found that the glandular epithelium cluster had the greatest expression of ERA genes, emphasizing the importance of this cellular compartment at the embryo-endometrium interface. Previous work has suggested that the molecular signature of the glandular epithelium correlates strongly with menstrual cycle day (69). Our results expand on this finding by suggesting that the importance of the glandular epithelium lies in its key role as the cell type driving receptivity in the mid-secretory endometrium. By identifying glandular epithelial markers of receptivity that are detectable in bulk mRNA-Seq, we generated a set of receptivity markers (the GERM signature), which was applied to published datasets and found to be altered in conditions suggestive of abnormal endometrial differentiation such as recurrent implantation failure and recurrent pregnancy loss. This novel signature therefore represents a promising new direction in the characterization of endometrial receptivity.

To better understand the epithelial contribution to receptivity, we calculated the GERM scores in published epithelial-only organoid cultures (59) and compared them with fertile endometrium datasets. This comparison revealed that while epithelial cells can generate a receptivity signal, full establishment of the receptive state likely requires stromal cell–derived signals, underscoring the importance of epithelial-stromal crosstalk in achieving optimal endometrial receptivity. Indeed, epithelial organoids appear less responsive to progestins than whole tissue (70), and epithelium isolated from endometrium tissue (GSE132711) scored higher than whole tissue (score = 7.1). Although the GERM score provides a promising framework for assessing receptivity, we acknowledge that this signature will require further refinement and independent validation in future studies to confirm its clinical utility. To accomplish this, a large number of well-characterized control samples from diverse populations, collected under strictly standardized conditions, may establish a baseline reference to which an individual patient’s mid-secretory biopsy could be compared.

Based on our data, we have proposed an important role for the glandular epithelium in defining endometrial differentiation and generating a receptivity signal. Understanding that endometrial differentiation is highly dependent on stromal cell decidualization as well, we next sought to examine this cellular compartment in greater depth. We compared endometrial gene expression with the gene expression patterns of in vitro stromal cells and uncovered key differences between these two models. Prior studies have examined the characteristics of stromal cells in vitro to gain a better understanding of the properties of these cells before and after the addition of a decidualization stimulus. In vitro decidualization is commonly carried out by culturing human endometrial stromal cells (HESCs) with one or more of the following stimuli: cAMP, medroxyprogesterone acetate, and estradiol (71). In these studies, decidualization is typically confirmed by appreciating changes in cellular morphology, from fibroblast-like to epithelioid, and assessing expression of markers including IGFBP1 and PRL (71). When we examined the expression of *IGFBP1* and *PRL* in our secretory phase and first trimester decidua samples, we found the highest expression of these markers in the pregnancy decidua, with relatively lower levels of expression in the secretory phase, suggesting that these markers may be used more aptly in models of early pregnancy decidua rather than to mimic the secretory phase endometrium in the absence of embryo implantation. To our knowledge, our study is one of the first to directly compare cell type–specific expression of these two markers and cellular morphology in both secretory phase biopsies and pregnancy decidua. Our data are in line with those that suggest that in the absence of pregnancy, the secretory phase can be thought of more aptly as a “pre-decidua” that only achieves full decidualization of stormal cells after embryo implantation (72). Our findings suggest the need for more complex in vitro assays to better assess the interactions between cells, as well as the need for a stimulus that more closely mimics the differentiation of the secretory-phase endometrium. In this way, the preimplantation endometrium may be studied ex vivo with higher fidelity.

Cellular senescence refers to the permanent proliferative arrest of a cell in response to various stressors (73). This process has been described in 2 forms: acute (transient and physiological) and chronic (persistent and age-related) (74, 75). Acute senescence may be observed as a part of normal processes, including embryogenesis, endometrial cycling, and repair of tissue injury (76, 77), whereas chronic senescence may represent the age-related or pathological decline in tissue function (76, 77). There is a growing appreciation for the role of senescence in endometrial remodeling (75, 78–82). Experimental evidence indicates that HESC decidualization is accompanied by the appearance of a p16-positive senescent cell subpopulation, suggesting that cellular senescence is a critical component of normal HESC decidualization (80). Embryo implantation may also activate physiological senescence (83). During decidualization, endometrial stromal cells undergo proliferation arrest and secrete inflammatory mediators, including senescence-associated secretory phenotype (82). Although decidual senescence is critical for the initial proinflammatory response required for embryo implantation (84), it is thought that premature senescence of human endometrial stromal cells can impair decidualization (79).

Within our 6 distinct stromal clusters, we resolved 3 clusters characterized by relative expression of decidualization marker *SCARA5* and senescence marker *DIO2*. Previous work has demonstrated an increase in senescent cells in the mid-secretory compared with the early secretory phase (69), as well as increased expression of *DIO2* and decreased expression of *SCARA5* in individuals with recurrent pregnancy loss compared with controls (44). We found that the highest levels of stroma-stroma communication occurred in the senescent clusters, followed by the decidualized clusters, and the lowest levels of signaling occurred in the senescent-decidualized cluster. We therefore hypothesized that the relative proportions of cells in each subgroup may vary in normal and pathological endometria and examined the expression patterns of *SCARA5* and *DIO2* in a published dataset that included women with recurrent implantation failure and controls. We found diminished expression of *DIO2* in women with recurrent implantation failure, as well as a larger proportion of cells in the senescent-decidualized cluster, suggesting that dysregulated senescence may contribute to, or be reflective of, suboptimal endometrial receptivity.

Our results highlight the dynamic crosstalk between stromal cells and the secretory glandular epithelium, particularly during the mid-secretory phase, underscoring the importance of these two cell types for endometrial receptivity. We observed that stromal-to-epithelial communication was highest in the mid-secretory phase, with the secretory glandular epithelium being the primary recipient of collagen-associated signaling. The ECM, particularly via collagen signaling, plays a fundamental role in embryo implantation by regulating endometrial tissue stiffness and mechano-sensing through receptors such as CD44 (85). Interestingly, CD44 expression is reduced in patients with infertility (86), though mouse models lacking CD44 remain viable and fertile (87), suggesting the presence of redundant receptors compensating for ECM-mediated signaling. This redundancy could be critical for maintaining implantation competence despite variations in individual receptor expression. The role of ECM mechanics in implantation is further supported by findings that collagenase-mediated softening of the ECM enhances fertility in mice (88), reinforcing the hypothesis that ECM remodeling influences implantation efficiency.

A limitation of our study is that it does not account for immune cell interactions, which are known to play a critical role in endometrial remodeling in preparation for embryo implantation. The maternal immune system modulates endometrial receptivity, and immune cells such as decidual macrophages and uterine NK cells are involved in ECM remodeling and trophoblast invasion. Recent studies highlighted the importance of immune-endometrial crosstalk in endometrium, showing dysregulated signaling in endometriosis, particularly epithelium to macrophage crosstalk through cytokine-mediated signaling pathways (89). Future studies should integrate immune cell populations into cell-cell interaction models to provide a more comprehensive understanding of implantation dynamics.

The importance of epithelial glands (59) in implantation has been well-documented across multiple species, including mice (29–31) and sheep (27, 28). Deletion of *Foxa2*, a glandular epithelium marker, demonstrated that the absence of this transcription factor led to defective uterine gland development (90) or function and subsequent implantation failure, underscoring the critical role of glandular secretions in establishing a receptive endometrial environment (91, 92). More recently, ESR1-dependent uterine gland structure has been shown to be critical for production of the key glandular secretion leukemia inhibitory factor (93). Further, in the mouse, uterine glands undergo a characteristic reorganization of the branched glands toward the implantation site prior to embryo attachment, supporting a dynamic role for the uterine glands in uterine receptivity (94). Similarly, studies in sheep indicate that endometrial glands produce key factors required for early pregnancy establishment, and that disruption of glandular function results in implantation failure (95). Taken together with our findings in the human endometrium, these data underscore the conserved and essential role of endometrial epithelial glands in implantation across species.

## Methods

### Sex as a biological variable.

This study exclusively involved female patients. The rationale for this is that the research focused on the dynamic changes in the human endometrium, a tissue unique to the female reproductive system, during the menstrual cycle and in early pregnancy. These are biological processes specific to females, and therefore the findings are not relevant to males.

### Supplemental methods.

Detailed descriptions of study participants, sample collection and processing, RNA-Seq workflows, and analytical methods are provided in the Supplemental Methods.

### Statistics.

Statistical analyses were conducted using the R statistical computing environment, with specific package versions cited in the relevant subsections. For bulk RNA-Seq, model-based differential expression analysis was conducted using the edgeR-robust method, which employs a negative binomial distribution. Correction for multiple hypothesis testing was performed using the Benjamini-Hochberg FDR method. Genes were considered differentially expressed if the FDR-corrected value was less than 0.05. *P* values of less than 0.05 were considered significant.

For scRNA-Seq data, feature expression measurements were normalized using a global-scaling normalization method (LogNormalize), which normalizes expression by total expression per cell, applies a scale factor of 10,000, and log-transforms the result. Dimensionality reduction was performed using principal component analysis on the top 2,000 variable genes; the top 20 principal components were selected for downstream clustering based on an elbow plot. Ligand-receptor communication probabilities were modeled using CellChat.

For the GERM analysis, gene set enrichment was calculated using the fgsea function. For external validation datasets, differential expression was assessed using GEO2R or DESeq2. To perform the meta-analysis across datasets, adjusted values were combined using the sum of logs method (Fisher’s method) via the sumlog function in the metap package.

### Study approval.

The use of human tissue specimens was approved by the IRB at Rutgers Health (Pro2018002041). All study participants provided signed written informed consent prior to enrollment.

### Data availability.

Bulk and scRNA-Seq data have been deposited in NCBI’s Gene Expression Omnibus (GEO GSE289073 and GSE290822, respectively). Any additional information required to reanalyze the data reported in this paper is available from the lead contact upon request.

## Author contributions

GWB and ENP were responsible for conceptualization, methodology, validation, formal analysis, data curation, writing the original draft, review and editing of the manuscript, and visualization. GWB and ENP contributed equally to this work. Authorship order among co–first authors was determined based on the extent of contributions to study conception, data analysis, and manuscript drafting, with GWB listed first. MP contributed to conceptualization, investigation, writing of the original draft, and visualization. QZ contributed to conceptualization, investigation, writing of the original draft, and methodology. RL contributed to conceptualization, writing of the original draft, and review and editing of the manuscript. KB, JGP, and PS contributed to investigation and writing of the original draft. RA contributed to conceptualization and review and editing of the manuscript. AC contributed to conceptualization, formal analysis, data curation, writing of the original draft, review and editing of the manuscript, visualization, and supervision. NCD was responsible for conceptualization, methodology, validation, formal analysis, data curation, writing of the original draft, review and editing of the manuscript, visualization, supervision, project administration, and funding acquisition.

## Funding support

This work is the result of NIH funding, in whole or in part, and is subject to the NIH Public Access Policy. Through acceptance of this federal funding, the NIH has been given a right to make the work publicly available in PubMed Central.

- Eunice Kennedy Shriver National Institute of Child Health and Human Development K99HD112539 and SRI/Bayer discovery innovation grant to ENP.
- NIH R01HD109152 to RA.
- NIH R01AI148695 to NCD.

## Supplementary Material

Supplemental data

Supplemental table 1

Supplemental table 2

Supplemental table 3

Supplemental table 4

Supplemental table 5

Supplemental table 6

Supplemental table 7

Supplemental table 8

Supporting data values

## Version 1 01/23/2026

Electronic publication
