# Supplementary material for: Single-cell mapping of human endometrium and decidua reveals epithelial and stromal contributions to fertility
Source: JCI Insight. 2026 Jan 23;11(2):e195254. doi: 10.1172/jci.insight.195254 (PMC12892900; doi:10.1172/jci.insight.195254)
Supplement: Supplemental data [file jciinsight-11-195254-s111.pdf]

Supplemental Methods

Human subjects

Self-identified Black and Hispanic women, aged 23-41 years, with regular, ovulatory menstrual cycles and at least one prior pregnancy were prospectively enrolled between November 2020 and December 2022. Study participants were consented for the review of their medical records and collection of blood and endometrial samples. Women with endocrine or autoimmune disorders, including antiphospholipid syndrome and polycystic ovary syndrome, or anatomic disorders of the reproductive tract, including hydrosalpinges or submucosal fibroids, were excluded. Women with a history of pregnancy complications, including recurrent pregnancy loss, preeclampsia or fetal growth restriction were also excluded. The demographic characteristics of participants are presented in **Table S1**.

Sample collection and processing

For the collection of endometrial tissue samples, study participants reported that they were not actively trying to conceive and had not used hormonal treatment in the 3 months prior to recruitment. Once enrolled, study participants were instructed to use condoms during sexual intercourse for the duration of their enrollment. Endometrial biopsies were performed with the Endocell®, a disposable endometrial tissue sampler (Wallach Surgical Devices, Trumbull, CT, USA) in the proliferative phase between cycle days 10-13 (n = 6) and during the early (n = 10), mid (n = 8), or late (n = 3) secretory phases, timed based on the urinary LH surge (early secretory = LH + 1-2 days, mid-secretory = LH + 8-9 days, late secretory = LH + 12-13 days). On the day of the endometrial biopsy, blood (5 mL) was collected in nonheparinized, serum separator tubes, allowed to clot, and centrifuged

at 4C. Serum estradiol (E2) and progesterone (P4) levels were determined (LabCorp, Raritan, NJ). Correct timing of biopsies was confirmed with serum E2 and P4 levels and endometrial histopathology as determined by two gynecological pathologists who were blinded to the timing of the tissue collection (1).

For collection of endometrial deciduae, women scheduled for an elective termination of an uncomplicated pregnancy in the first trimester at 6-8 weeks gestation were eligible for inclusion. Study participants (n = 3) were recruited and enrolled prior to their scheduled surgical procedure. After dilation and curettage, tissue samples were floated in phosphate buffered saline (PBS, pH 7.2) and the endometrial decidua was isolated from the gestational sac and chorionic villi by mechanical separation using forceps.

After collection, endometrial tissue was placed in a tissue culture dish and rinsed with ice-cold PBS to remove blood and mucus. Using fine forceps, each sample was separated such that approximately 100 mg endometrial tissue was fixed in formalin and stored at room temperature for immunohistology; approximately 100 mg was preserved in RNAprotect (Qiagen, #76160) and stored at -80ºC for RNA extraction for bulk RNA sequencing; and the remaining tissue was placed on ice, minced and processed within one hour of collection for scRNA-Seq.

Isolation of endometrial and decidual cells

Cells were isolated from minced endometrial tissues for scRNA-Seq by incubation at 37°C in digestion media (DMEM/F12, 3% charcoal-stripped fetal bovine serum (FBS), 1 mg/mL collagenase A (Roche, #11088793001), 0.1 mg/mL DNAse type I (Roche, #10104159001) with agitation on a benchtop shaker at 250 rpm for 15 minutes. The tissue was then further

dissociated by repeatedly passing it through a 16G needle attached to a 10 mL syringe. The shaking and needle passage steps were repeated once more to ensure thorough dissociation. The cell suspension was centrifuged at 300× g for 5 minutes. The resulting pellet was resuspended in 1 mL of TrypLE Select Enzyme (Thermo Fisher #12563011) containing 0.1 mg/mL DNase I and incubated at 37°C on a shaker at 250 rpm for 15 minutes. 10 mL of ice-cold digestion media was added, and the cell suspension was filtered through a 40 μm cell strainer. Red blood cells (RBC) were lysed using RBC lysis buffer (ThermoFisher, #00-4333-57) and dead cells were removed using the Dead Cell Removal Kit (Miltenyi Biotec, #130-090-101). The resulting single-cell suspension was used for Chromium (10X Genomics) sequencing.

RNA isolation, library preparation, and sequencing

Total RNA was isolated from endometrial tissue using a RNeasy mini kit (Qiagen, #74136). RNA was quantified by Qubit (Invitrogen) and quality was assessed with a Fragment Analyzer (Advanced Analytical Technologies, Inc.) at Albert Einstein College of Medicine Epigenomics Shared Facility (RRID:SCR_023284). Libraries were prepared using the KAPA Stranded RNA-Seq Kit with RiboErase for Illumina Platforms (Kapa Biosystems #KK8483) with the addition of Ambion External RNA Controls Consortium (ERCC) spike-in controls (Invitrogen #4456740). Libraries were quantified, multiplexed, and sequenced as single end (1 x 75 bp) on an Illumina NextSeq 500 instrument (RRID:SCR_014983) to yield approximately 50 million reads per sample. FASTQ files were generated using picard (v2.26.10, RRID:SCR_006525) module ExtractIlluminaBarcodes followed by IlluminaBasecallsToFastq with default parameters, except "INCLUDE_NON_PF_READS = false". Raw FASTQ files were trimmed of flanking

adapter sequences using Trim Galore (v0.6.7, RRID:SCR_011847)(2) with default parameters and "adapter = AGATCGGAAGAGC". Read quality was assessed using FastQC (v0.11.9, RRID:SCR_014583) and FastQ Screen (v0.6.5, RRID:SCR_000141).

For scRNA-Seq, libraries were generated and sequenced using the 10X Chromium Single Cell 3' GEM kit (10X Genomics, v2). Paired end, 2 x 75 bp, sequencing was performed on an Illumina NextSeq 500 instrument (RRID:SCR_014983). Output was demultiplexed and converted to FASTQ format with Cell Ranger (10X Genomics, v3.1.0).

RNA sequencing analysis

Trimmed reads were mapped to Homo sapiens genome assembly GRCh38 (hg38) using STAR (v2.7.9a, RRID:SCR_004463)(3). Reads overlapping Ensembl (4) annotations (v110) were quantified with STAR prior to model-based differential expression analysis using the edgeR-robust method (5-7). A read counts matrix was used as input for the endest (8) R package (v0.1.1) results were visualized with ggplot2 (v3.5.1, RRID:SCR_014601). For differential expression analysis, genes with low counts per million (CPM) were removed using the filterByExpr function from edgeR (RRID:SCR_012802). Genes were considered differentially expressed if the FDR-corrected p-values were less than 0.05. Venn diagrams were generated with the R package eulerr (v7.0.2, RRID:SCR_022753).

For scRNA-Seq, demultiplexed sequencing reads were processed and aligned to the *Homo sapiens* genome assembly GRCh38 (hg38) using STAR (v2.7.9a) with 10X Genomics Cell Ranger (v3.1.0, RRID:SCR_017344). Samples were merged using the integration anchors function of the Seurat package (v5.1.0, RRID:SCR_016341) in R (9). Genes expressed in fewer than three cells in a sample were excluded, as well as cells

that expressed fewer than 200 genes and mitochondrial gene content >5% of the total unique molecular identifier count. Data were normalized using a global-scaling normalization method (9) that normalizes the feature expression measurements for each cell by the total expression, multiplies this by a scale factor (10,000), and then log-transforms the results. High *HLA-G* and *CGA* cells were identified in pregnant biopsies as trophoblast were removed from our analysis. The top 2,000 most variable genes that were used for cell clustering were found using the *FindVariableFeatures* function and were then normalized using the *ScaleData* function. Based on an elbow plot generated using the *Elbowplot* function of Seurat, we selected 20 principal components (PC) for downstream analyses. Cell clusters were generated using *FindNeighbors* and *FindClusters* functions. For visualization, UMAPs were generated using the *RunUMAP*, *FeaturePlot* and *DimPlot* functions. The *DotPlot* Seurat function was used to generate dot plots to visualize gene expression for each assigned cluster. The Seurat function *AddModuleScore* was used to calculate ERA and decidualization scores. Ligand-receptor cellular communication analysis was determined with the CellChat (v2.1.2, RRID:SCR_021946) (10, 11) R package. Briefly, communication probabilities were calculated between cell types using the functions identifyOverExpressedGenes and identifyOverExpressedInteractions. Signaling sources, influencers, targets, mediators, and high-order information were obtained by communication network analysis. The resulting interactions were visualized using the included plotting functions.

Glandular Epithelium Receptivity Module (GERM) score calculation

The receptivity module signature genes (n = 556, Table S7) were separated by positive or negative fold change and described as “GERM_up” or “GERM_down”, respectively,

where "GERM_up" genes were increased at mid-secretory phase. The resulting dataframe was used as input for *fgsea* (12) enrichment analysis implementation in clusterProfiler (v4.12.6, RRID:SCR_016884) (13) with multiple datasets. Microarray and bulk RNA-Seq experiment results were downloaded from NCBI GEO (RRID:SCR_005012), using the GEO2R tool, as a list of genes and fold changes for early versus mid-secretory endometrium. Human endometrial epithelial organoid culture data (14) were not available for evaluation with GEO2R; therefore, raw read counts were downloaded from GEO and analyzed with DESeq2 (v1.48.2, RRID:SCR_015687) (15) using the same parameters. Spatial and scRNA-Seq datasets were subset for the glandular or secretory glandular epithelium cell types. A combined normalized enrichment score (NES) was calculated as $GERM\ score = GERM\_up + -1\ (GERM\_down)$ and adjusted p-values were combined using the metap package with the *sumlog* function. The results were compiled and then visualized as a dotplot with ggplot2 (v3.5.1, RRID:SCR_014601).

Immunostaining and Imaging

Formalin-fixed and paraffin-embedded endometrial tissue was sectioned at 5 mm, placed on charged glass slides (StatLab, Millenia 1000), air-dried and stored at room temperature before use. Sections were stained with hematoxylin and eosin (H&E) or immunohistochemistry (IHC) staining. For IHC, sections were deparaffinized followed by antigen retrieval with BOND Epitope Retrieval Solution 2 (Leica Biosystems, USA) for 40 minutes. Primary antibodies to detect the estrogen receptor alpha (clone 6F11, Leica Biosystems) and progesterone receptor (clone 16, Leica Biosystems) were applied for 20 and 24 minutes, respectively. Bound primary antibodies were detected with BOND

Polymer Refine Detection kit. Sections were washed with distilled water, counterstained with hematoxylin, dehydrated through graded alcohols, cleared in xylene, and mounted with synthetic permanent media.

References

1. Chemerinski A, Shen M, Valero-Pacheco N, Zhao Q, Murphy T, George L, et al. The impact of ovarian stimulation on the human endometrial microenvironment. *Hum Reprod.* 2024;39(5):1023-41.

2. Martin M. Cutadapt removes adapter sequences from high-throughput sequencing reads. *EMBnetjournal.* 2011;17(1):pp-10.

3. Dobin A, Davis CA, Schlesinger F, Drenkow J, Zaleski C, Jha S, et al. STAR: ultrafast universal RNA-seq aligner. *Bioinformatics.* 2013;29(1):15-21.

4. Aken BL, Ayling S, Barrell D, Clarke L, Curwen V, Fairley S, et al. The Ensembl gene annotation system. *Database (Oxford).* 2016;2016:1-19.

5. Robinson MD, McCarthy DJ, and Smyth GK. edgeR: a Bioconductor package for differential expression analysis of digital gene expression data. *Bioinformatics.* 2010;26(1):139-40.

6. McCarthy DJ, Chen Y, and Smyth GK. Differential expression analysis of multifactor RNA-Seq experiments with respect to biological variation. *Nucleic Acids Res.* 2012;40(10):4288-97.

7. Zhou X, Lindsay H, and Robinson MD. Robustly detecting differential expression in RNA sequencing data using observation weights. *Nucleic Acids Res.* 2014;42(11):e91.

8. Agarwal V, Bell GW, Nam JW, and Bartel DP. Predicting effective microRNA target sites in mammalian mRNAs. *Elife.* 2015;4:e05005.

9. Hao Y, Hao S, Andersen-Nissen E, Mauck WM, 3rd, Zheng S, Butler A, et al. Integrated analysis of multimodal single-cell data. *Cell.* 2021;184(13):3573-87.e29.

10. Jin S, Guerrero-Juarez CF, Zhang L, Chang I, Ramos R, Kuan CH, et al. Inference and analysis of cell-cell communication using CellChat. *Nat Commun.* 2021;12(1):1088.

11. Jin S, Plikus MV, and Nie Q. CellChat for systematic analysis of cell-cell communication from single-cell transcriptomics. *Nat Protoc.* 2024.

12. Korotkevich G, Sukhov V, Budin N, Shpak B, Artyomov MN, and Sergushichev A. Fast gene set enrichment analysis. *bioRxiv.* 2021:060012.

13. Wu T, Hu E, Xu S, Chen M, Guo P, Dai Z, et al. clusterProfiler 4.0: A universal enrichment tool for interpreting omics data. *Innovation (Camb).* 2021;2(3):100141.

14. Fitzgerald HC, Dhakal P, Behura SK, Schust DJ, and Spencer TE. Self-renewing endometrial epithelial organoids of the human uterus. *Proc Natl Acad Sci U S A.* 2019;116(46):23132-42.

15. Love MI, Huber W, and Anders S. Moderated estimation of fold change and dispersion for RNA-seq data with DESeq2. *Genome Biol.* 2014;15(12):550.

Supplemental Figures

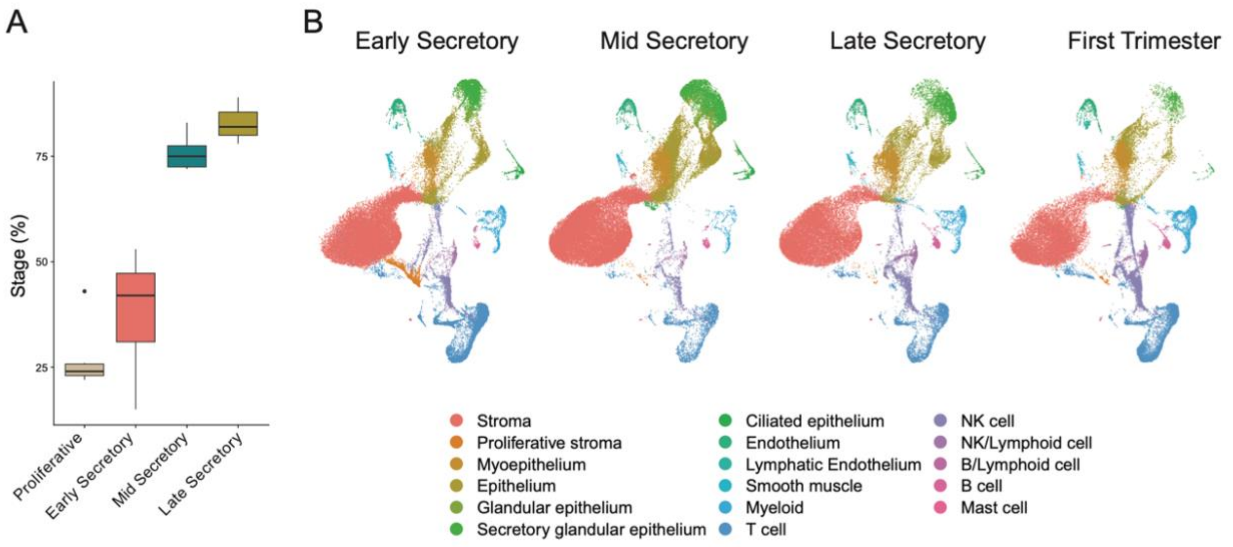

Figure S1. Additional information related to menstrual cycle phase. (**A**) Boxplot of sample staging from bulk mRNA-seq (*n* = 26) using the eldest package from Teh and colleagues (33). (**B**) Uniform Manifold Approximation and Projection (UMAP) visualization of scRNA-Seq data from endometrial biopsies from the early (*n* = 4), mid- (*n* = 4), and late secretory (*n* = 3) phases, and first trimester decidua samples (*n* = 3).

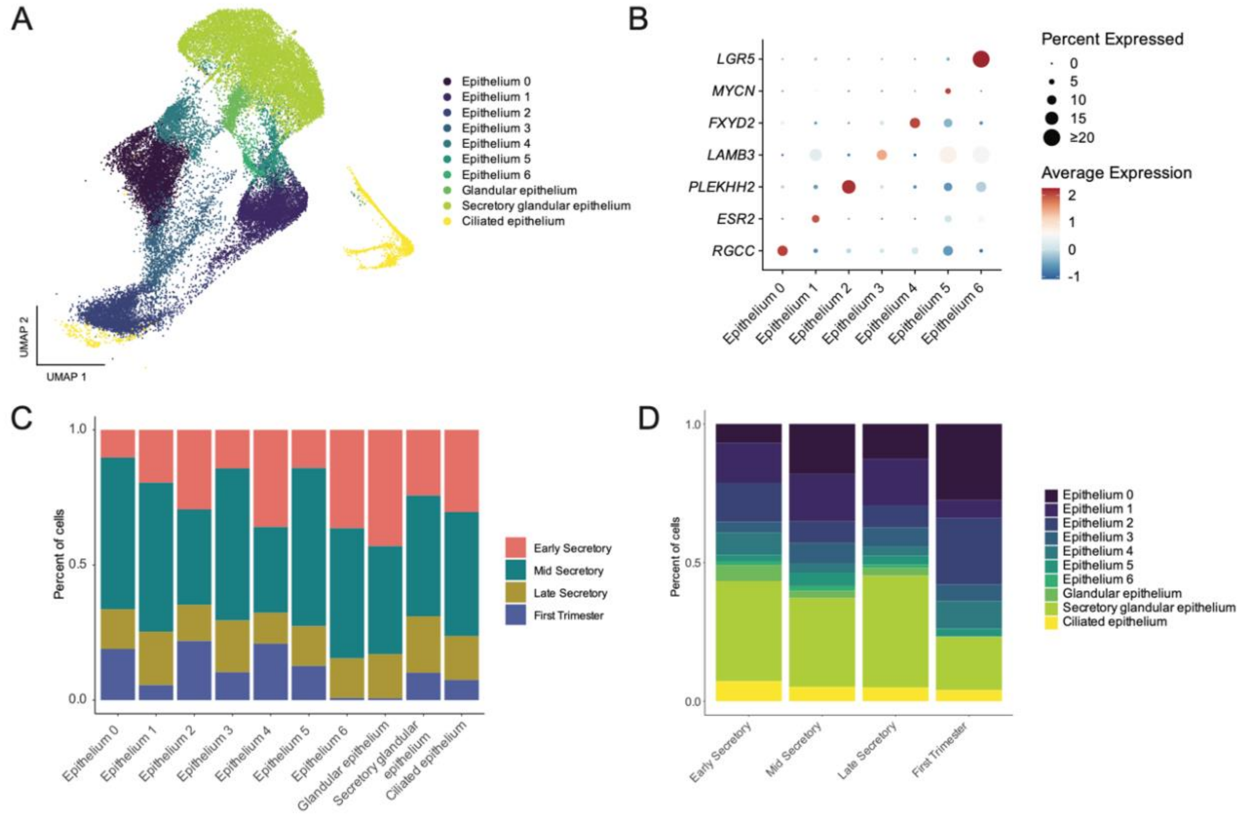

Figure S2. Epithelium subclusters and distribution across cycle stages. (**A**) UMAP visualization of scRNA-Seq data from human epithelium sub-clusters. (**B**) Dot plot of marker genes for each sub cluster. (**C**) Stacked bar plots showing the contribution of each cycle stage to the epithelial sub-clusters and (**D**) the proportion of epithelial sub-clusters split by stage.

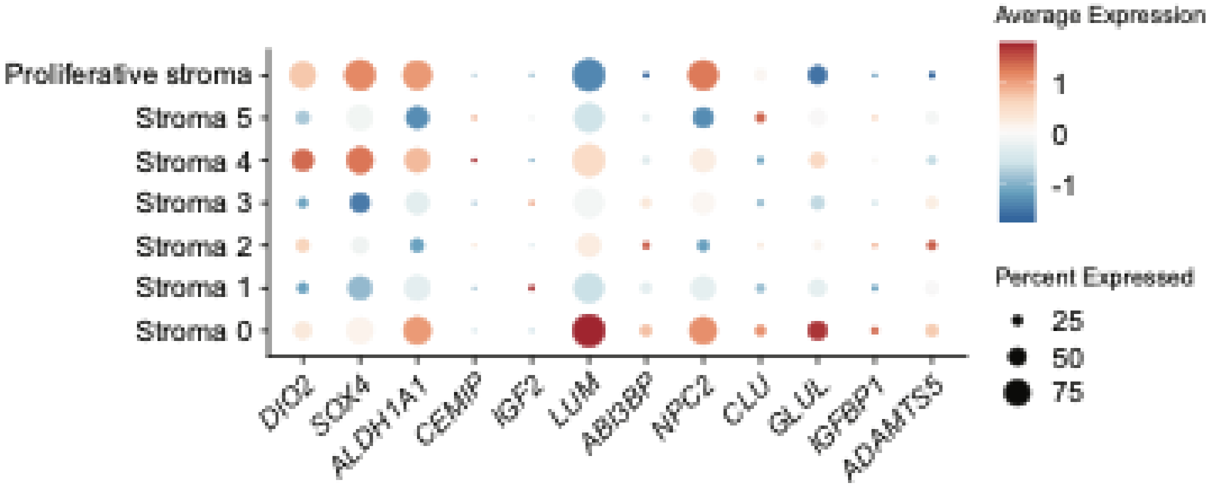

Figure S3. Expression of in vitro senescence markers is not restricted to a specific stroma subcluster. Dotplot of in vitro senescence markers in the stroma sub-clusters.
